# Transcriptomics and network pharmacology reveal the potential mechanism related to integrated stress response in the treatment of osteoporosis by Jiawei Shentong Zhuyu Decoction and verified by RT-qPCR

**DOI:** 10.3389/fendo.2025.1618296

**Published:** 2025-10-21

**Authors:** Jie Zhao, TingTing Zhang, Pengbo Han, Zhu Liu, Hongli Zhang, Fangqin Li, Gang Han

**Affiliations:** ^1^ Traditional Chinese Medicine Department, The Second Affiliated Hospital of Xi’an Medical College, Xi’an, Shaanxi, China; ^2^ Department of Rheumatology and Immunology, Xi’an Fifth Hospital, Xi’an, Shaanxi, China

**Keywords:** osteoporosis, Jiawei Shentong Zhuyu Decoction, integrated stress response, *COL4A6/NKX3-1*, network pharmacology, quercetin

## Abstract

**Background:**

Osteoporosis (OP) is a systemic skeletal disorder. The molecular mechanisms underlying the effects of Jia Wei Shentong Zhuyu Decoction (JWSTZYD) and the integrated stress response (ISR) in OP remain unclear. This study aims to elucidate the mechanisms by which JWSTZYD and ISR contribute to OP.

**Methods:**

Potential drug target genes for JWSTZYD and OP-related datasets were sourced from the Gene Expression Omnibus (GEO) and Traditional Chinese Medicine Systems Pharmacology (TCMSP) databases. Differentially expressed genes (DEGs) in OP were identified by analyzing transcriptome data. Candidate genes were selected by intersecting target genes, DEGs, and ISR-related genes (ISR-RGs), with further screening based on expression levels in OP and control samples. The potential mechanisms of these biomarkers in OP were explored through gene set enrichment analysis (GSEA), immune infiltration analysis, molecular regulatory networks, and molecular docking. Expression levels of biomarkers were validated using clinical samples.

**Results:**

*COL4A6* and *NKX3–1* were identified as biomarkers associated with ISR in JWSTZYD treatment of OP. These biomarkers were significantly enriched in 22 and 23 pathways, respectively. Immune infiltration analysis revealed 10 differentially abundant immune cell types between OP and control. Further analysis showed that 16 transcription factors (TFs), 26 miRNAs, and 342 lncRNAs had potential interactions with the biomarkers. TFs such as FOXC1, USF2, NFYA, SRF, and NFIC were co-regulated by the biomarkers. Quercetin was identified as a drug that co-acted with these biomarkers, demonstrating strong binding affinity with both *COL4A6* and *NKX3-1*, with binding energies of -6.3 kcal/mol and -15.7 kcal/mol, respectively. Experimental validation confirmed that the biomarkers were expressed at levels consistent with those predicted.

**Conclusion:**

This study identified *COL4A6* and *NKX3–1* as key biomarkers, providing new insights into the mechanisms associated with ISR in the treatment of OP using JWSTZYD.

## 1 Introduction

Osteoporosis (OP) is a systemic bone disorder characterized by the deterioration of bone tissue and reduced bone mineral density. The etiology of OP is classified into primary and secondary forms. Primary OP is typically observed as postmenopausal or senile OP, while secondary OP often results from conditions such as glucocorticoid-induced OP or diabetes (1, 2). The burden of OP significantly affects the quality of life, with fractures being the most severe complication. Due to the aging population, OP is considered a major public health issue, imposing a substantial socio-economic burden (3).

Given the complex regulatory mechanisms governing bone homeostasis, a variety of pharmaceutical agents targeting OP have been introduced into clinical practice. These include bisphosphonates, calcitonin, selective estrogen receptor modulators, and molecular-targeted drugs. However, many of these drugs are associated with considerable adverse effects or are unsuitable for long-term use. As a result, there is an urgent need for the development of more effective therapeutic options for OP (4).

The Shentong Zhuyu Decoction (STZYD) is an established traditional formula that is effective in treating orthopedic diseases (5–8). Jia Wei Shentong Zhuyu Decoction (JWSTZYD) is a modified version of STZYD, with the addition of Dipsaci Radix and Cibotii Rhizoma, two herbs commonly used in the treatment of OP, demonstrating significant clinical efficacy (9, 10). Recent pharmacological studies have shown that JWSTZYD inhibits the inflammatory response, alleviates pain, improves blood circulation, and promotes the resolution of blood stasis (11, 12). From a mechanistic perspective, Dipsaci Radix can enhance bone density and regulate the differentiation pathways of osteoclasts (13–15). Cibotii Rhizoma enhances bone formation by upregulating osteoprotegerin expression and downregulating *RANKL* expression in osteoblasts and bone marrow stromal cells (16). However, the precise mechanism of JWSTZYD in treating OP remains unclear.

Network pharmacology (NP) is a comprehensive field that seeks to elucidate the therapeutic mechanisms of drugs in disease treatment through the identification of biological targets and pathways (17). This approach is particularly suitable for studying the pharmacological mechanisms of traditional Chinese medicine formulas due to its systematic and holistic nature (18). Extensive research has already utilized network pharmacological methods to explore the pharmacological mechanisms of traditional Chinese medicine formulas (19, 20). The Integrated Stress Response (ISR) refers to signaling pathways activated by stress-induced eIF2α kinases (21), which enable cells, tissues, and organisms to adapt to environmental changes and maintain homeostasis (22). From a medical perspective, the ISR is implicated in the pathogenesis of various disorders, and manipulating the ISR has emerged as a promising therapeutic strategy for several diseases (23).

Osteoporosis (OP), as a common metabolic bone disease, requires further exploration of its prevention and treatment strategies. JWSTZYD has demonstrated potential for improving bone metabolism in clinical applications, yet its mechanism of action remains incompletely elucidated. NP provides a systematic approach to deciphering the multi-component, multi-target mechanisms of traditional Chinese medicine formulas. The integrated stress response (ISR), a key pathway regulating cellular homeostasis, has been implicated in the pathogenesis of various skeletal disorders (2, 24).However, its role in JWSTZYD treatment for OP has not been previously reported.

Based on this, the core hypothesis of this study is proposed: JWSTZYD may exert therapeutic effects on OP by regulating bone metabolic balance (promoting bone formation and inhibiting bone resorption) and the immune microenvironment through modulating ISR-related targets (e.g., key genes and signaling pathways). We further hypothesize that key active components in JWSTZYD may bind to core ISR-related genes to activate or inhibit downstream bone metabolism-related signaling pathways, forming the crucial molecular basis for its action.

To validate this hypothesis, this study screened for potential biomarkers associated with ISR in JWSTZYD-treated OP by analyzing expression level differences and trends between OP and control groups using transcriptome data and drug target data from public databases. Concurrently, through comprehensive bioinformatics analysis, we delved into the biological functions, immune cell infiltration characteristics, and molecular regulatory networks associated with these biomarkers. Molecular docking validation confirmed the binding affinity between key active components and their targets. These findings provide novel theoretical foundations and experimental evidence elucidating the mechanism by which JWSTZYD treats OP through ISR regulation.

## 2 Materials and methods

### 2.1 Data collection

**Figure 1:**
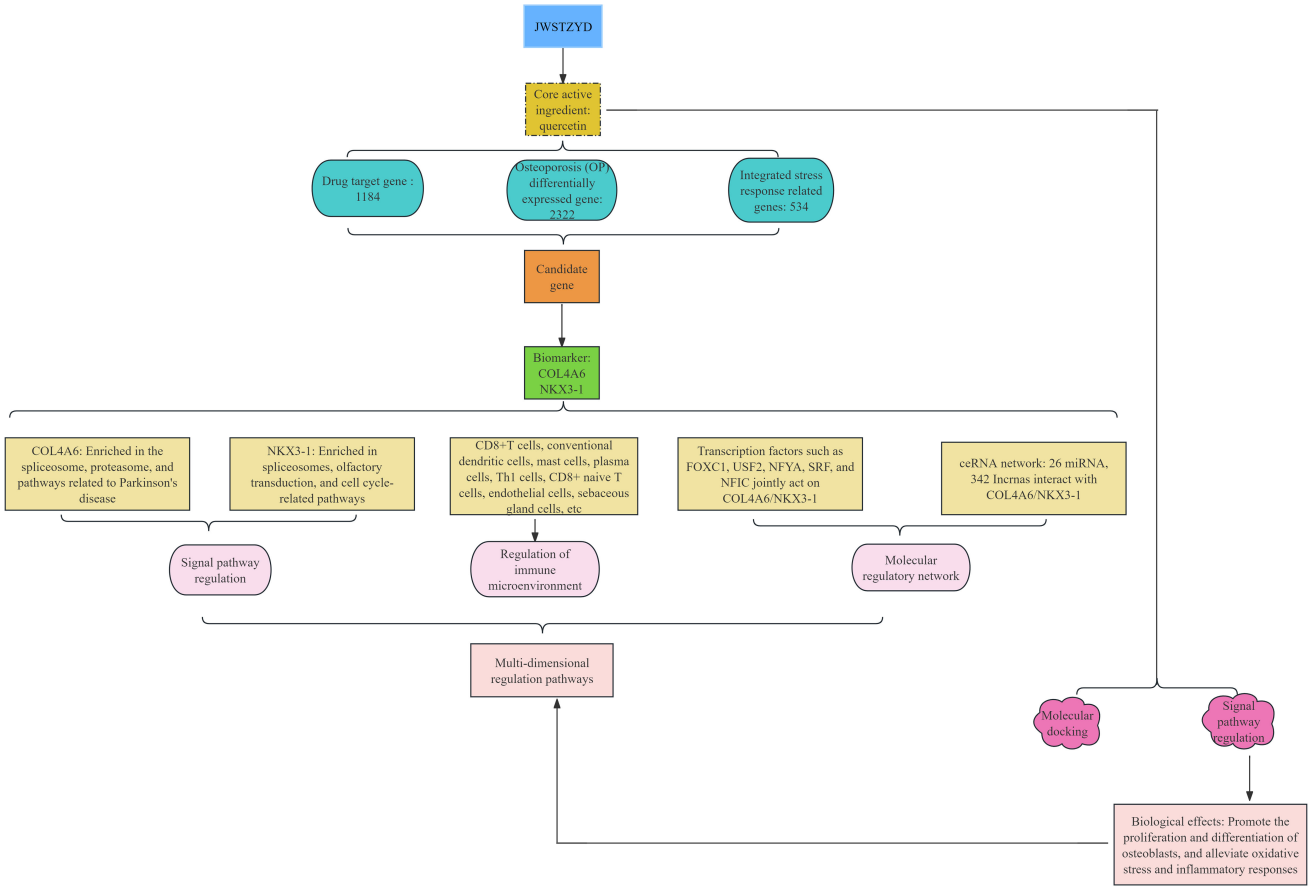
Flowchart of this study.

The overall design and analytical workflow of this study is summarized in **Figure 1**. First, the required data were obtained from public databases. The OP-related transcriptome data (GSE56815 and GSE230665) were retrieved from the Gene Expression Omnibus (GEO) database (https://www.ncbi.nlm.nih.gov/geo/). GSE56815 (GPL96) served as the training set, comprising 40 low bone mineral density (L-BMD) samples (OP) and 40 high bone mineral density (H-BMD) samples (control). GSE230665 (GPL10332) was used as the validation set, containing 12 OP samples and 3 control samples. A total of 534 ISR-related genes (ISR-RGs) were sourced from relevant literature (25) (**Supplementary Table 1**).

### 2.2 Screening of active ingredients and drug targets

JWSTZYD primarily consists of Gentiana macrophylla (Qinjiao), Hansenia weberbaueriana (Qianghuo), Prunus persica (Taoren), Carthamus tinctorius (Honghua), Angelica sinensis (Danggui), Ligusticum sinense (Chuanxiong), Commiphora myrrha (Moyao), Ganoderma lucidum (Lingzhi), Cyperus rotundus (Xiangfu), Achyranthes bidentata (Niuxi), Pheretima (Dilong), Glycyrrhiza uralensis (Gancao), Dipsacales (Xuduan), and Woodwardia japonica (Gouji). The main active ingredients and drug target points of JWSTZYD were screened through the Traditional Chinese Medicine Systems Pharmacology (TCMSP) database (https://old.tcmsp-e.com/tcmsp.php), using thresholds for Oral Bioavailability (OB ≥ 30%) and Drug-Likeness (DL ≥ 0.18). Drugs not found in the TCMSP database were searched through the HERB database. The potential target genes were obtained by standardizing target points in the UniProt database (https://www.uniprot.org). A network of traditional Chinese medicines, active ingredients, and target genes was constructed using Cytoscape software (v 3.7.2).

### 2.3 Identification of differential expression genes

DEGs between L-BMD and H-BMD samples in GSE56815 were identified using the “limma” package (v 3.54.0) (26) (P < 0.05). The top 10 genes with the greatest upregulation and downregulation were selected based on log_2_FC values. A volcano plot was generated using the “ggplot2” package (v 3.4.4) (27), highlighting the DEGs and the top 10 up- and down-regulated genes. A heatmap was created using the “ComplexHeatmap” package (v 2.14.0) (28) to display the top 10 up- and down-regulated genes between L-BMD and H-BMD samples.

### 2.4 Identification, enrichment analysis, and subcellular localization of candidate genes

To identify candidate genes associated with JWSTZYD and ISR-RGs in L-BMD, this study intersected the DEGs, ISR-RGs, and target genes of JWSTZYD using the “VennDiagram” package (v 1.7.3) (29). Gene Ontology (GO) and Kyoto Encyclopedia of Genes and Genomes (KEGG) enrichment analyses were conducted to explore the biological pathways and functions of candidate genes using the “clusterProfiler” package (v 4.7.1.003) (30). GO analysis was used to examine the molecular functions (MF), biological processes (BP), and cellular components (CC) of genes, with the top 5 pathways from each section presented based on P-value sorting (P < 0.05). KEGG analysis was performed to identify the most significant metabolic and signal transduction pathways (P < 0.05), and the top 15 pathways were displayed. To explore the subcellular localization of candidate genes, subcellular localization analysis was performed using the mRNALocater database (http://bio-bigdata.cn/mRNALocater/). The results were visualized in a bar chart generated using the “ggplot2” package (v 3.4.4).

### 2.5 Expression analysis

Candidate gene expression differences and trends between L-BMD and H-BMD samples in both the training and validation sets were utilized for further gene screening. Expression differences were assessed using the Wilcoxon test from the “rstatix” package (v 0.7.2) (https://CRAN.R-project.org/package=rstatix) (P < 0.05), and the results were visualized using the “ggplot2” package (v 3.4.4). Genes that exhibited significant differences and consistent trends across both datasets were selected as biomarkers for subsequent analysis.

### 2.6 Enrichment analysis of biomarkers

Gene set enrichment analysis (GSEA) was employed to investigate the biological pathways associated with the biomarkers in OP. The reference set used was “c2.cp.kegg.v7.4.symbols.gmt” from the MSigDB Database (www.gsea-msigdb.org/gsea/msigdb). Based on the biomarker expression levels in the samples, GSE56815 was divided into high/low expression groups for differential analysis. The fold change (FC) of DEGs was sorted from largest to smallest. GSEA was performed using the “clusterProfiler” package (v 4.7.1.003), and the top 5 pathways were displayed (P < 0.05).

### 2.7 Immune infiltration analysis

Immune cell infiltration in OP was analyzed using immune infiltration analysis. The infiltration of 64 immune cell types (31) between high and low BMD was assessed using the Xcell algorithm from the “IOBR” package (v 0.99.8) (32). Differences in immune cells between the OP and control groups were tested using the Wilcoxon test (P < 0.05), and the results were visualized with the “ggpubr” package (v 0.6.0) (33). Correlations between differential immune cells and biomarkers were analyzed using the “stats” package (v 4.3.1) (34). A correlation coefficient (cor) > 0.3 and P < 0.05 indicated a strong association between differential immune cells and biomarkers. The correlation heatmap was generated using the “ggcor” package (v 0.9.8.1) (35).

### 2.8 Prediction of targeted molecules

The interactions between lncRNAs, TFs, and miRNAs with biomarkers were predicted using relevant databases to explore the molecular regulatory mechanisms of biomarkers. TFs were predicted using the JASPAR database (https://jaspar.genereg.net/) from the NetworkAnalyst platform. MiRNAs were predicted using the DIANA MicroT database (http://mirtoolsgallery.tech/mirtoolsgallery/node/1084) and the ELMMo database (http://mirtoolsgallery.tech/mirtoolsgallery/node/1098) *via* the “multiMiR” package (v 0.98.0.2) (36). The intersection results of these two databases were analyzed further. The miRNet database was used to predict lncRNAs interacting with miRNAs. The TF-mRNA and lncRNA-miRNA-mRNA regulatory networks were visualized using Cytoscape software (v 3.7.2).

### 2.9 Molecular docking

To further explore the relationship between biomarkers and drugs, molecular docking was conducted between potential drugs and biomarkers. Biomarker targets were retrieved from the UniProt database (http://www.uniprot.org), followed by the identification of active drugs interacting with these targets. Drugs acting on two biomarker targets were analyzed as key drugs. The sdf files for these key drugs were obtained from the PubChem database (http://pubchem.ncbi.nlm.nih.gov). Corresponding protein structures for the biomarkers were sourced from the PDB database (https://www.rcsb.org/) and the AlphaFold Protein Structure database (http://alphafold.ebi.ac.uk). The protein structures of the biomarkers were subjected to molecular docking with the active ingredients using the CB-Dock2 database (https://cadd.labshare.cn/cb-dock2/php/index.php), and binding energies were calculated. A binding energy ≤ -5 kcal/mol was considered indicative of strong binding affinity. Molecular docking results were visualized using the “pymol” package (v 3.0.3) (37).

### 2.10 Clinical sample validation

The expression differences of biomarkers between L-BMD (OP) and H-BMD (control) were validated by RT-qPCR. A total of 5 pairs of whole blood samples were collected from the Second Affiliated Hospital of Xi’an Medical College, including 5 OP and 5 control samples. All participants signed informed consent forms, and the study was approved by the hospital’s ethical review board (Approval Number: X2Y2025007S). Total RNA from the blood samples was extracted using TRIzol reagent (Ambion, USA), and RNA concentrations were measured using the NanoPhotometer N50. Subsequently, mRNA was reverse transcribed into cDNA using a test kit (Servicebio, Wuhan, China). RT-qPCR was performed, with detailed reaction mixtures, primer sequences, and experimental conditions provided in **Supplementary Table 2**. Biomarker expression levels were quantified using the 2^-ΔΔCt^ method, with *GAPDH* as the internal reference gene for normalization. Data were analyzed with GraphPad Prism 5, and P-values were calculated. Expression differences between OP and control samples were evaluated using a t-test (P < 0.05).

### 2.11 Statistical analysis

Bioinformatics analyses were performed using R (v 4.3.1), with group differences assessed *via* the Wilcoxon test. Statistical significance was defined as P < 0.05. Expression differences in RT-qPCR experiments between OP and control samples were also evaluated using a t-test (P < 0.05).

## 3 Results

### 3.1 Construction of drug, active ingredient and target network, and acquisition of DEGs

**Figure 2:**
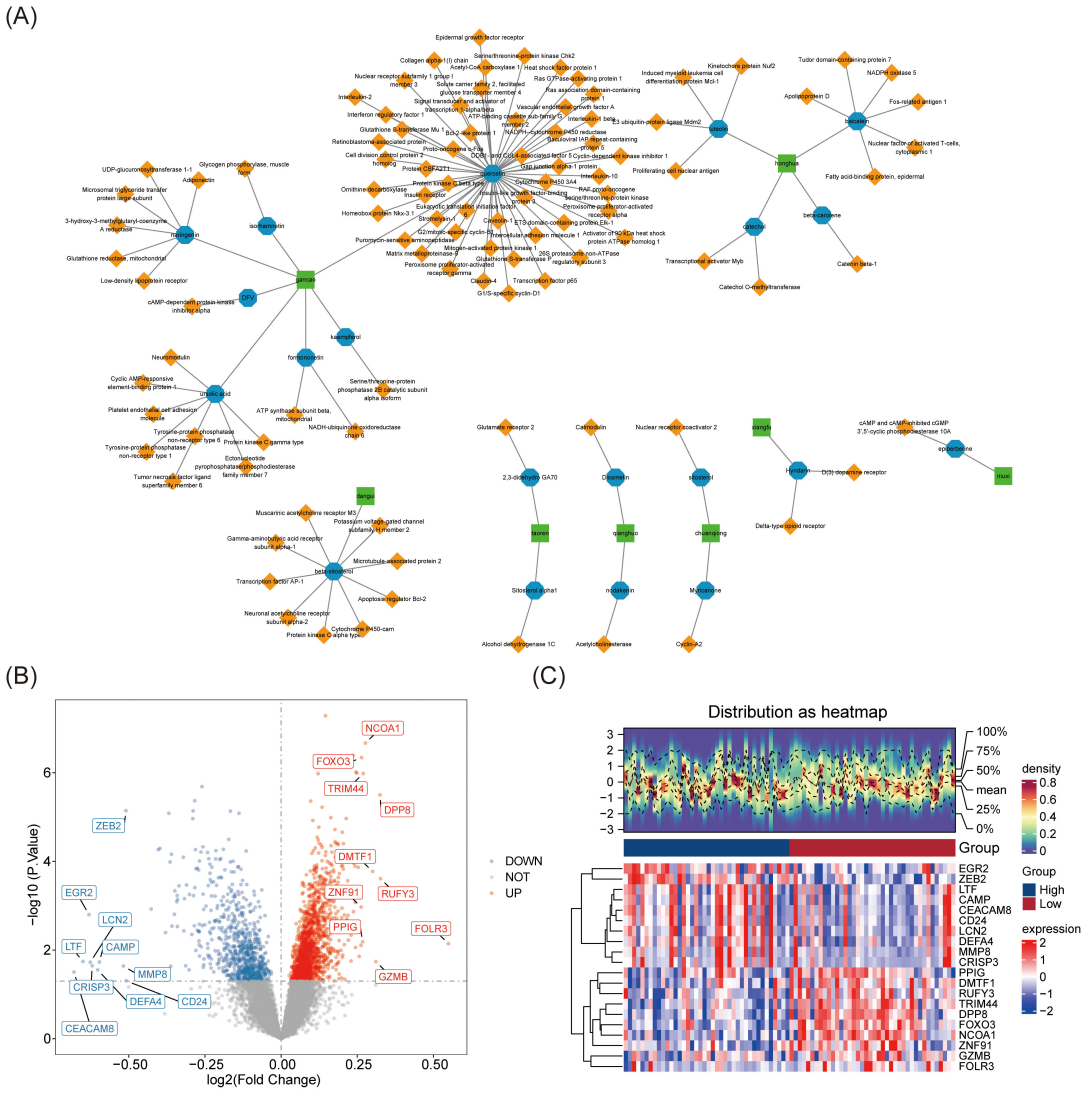
Screening of the main active compounds and their targets of JWSTZYD and differential expression analysis in the training set. **(A)** Traditional Chinese Medicine - Active Ingredients - Target Network: green represents traditional Chinese medicinal materials, blue represents active compounds, and yellow represents drug action targets. **(B)** Volcano plot of differential expression analysis of genes (DEGs) in Osteoporosis (OP): red dots represent up-regulated genes, and blue dots represent down-regulated genes. **(C)** Heatmap of DEGs differential expression analysis in OP.

The 14 Chinese herbal ingredients and 299 active compounds of JWSTZYD were screened through the database (**Supplementary Table 3**), leading to the identification of 1,184 target genes (**Supplementary Table 4**). The network of Chinese herbal ingredients, active ingredients, and target genes revealed that Gancao was associated with the most active ingredients, while Quercetin (QUE) interacted with the most target genes (**Figure 2A**).

In the training set, 2,322 DEGs were identified, including 774 down-regulated genes and 1,548 up-regulated genes (**Supplementary Table 5**). Based on the log_2_FC values, the top 10 up- and down-regulated genes were displayed in a volcano plot (**Figure 2B**). A heatmap visualized the top 10 DEGs between the L-BMD and H-BMD groups (**Figure 2C**).

### 3.2 Biological function analysis of candidate genes

**Figure 3:**
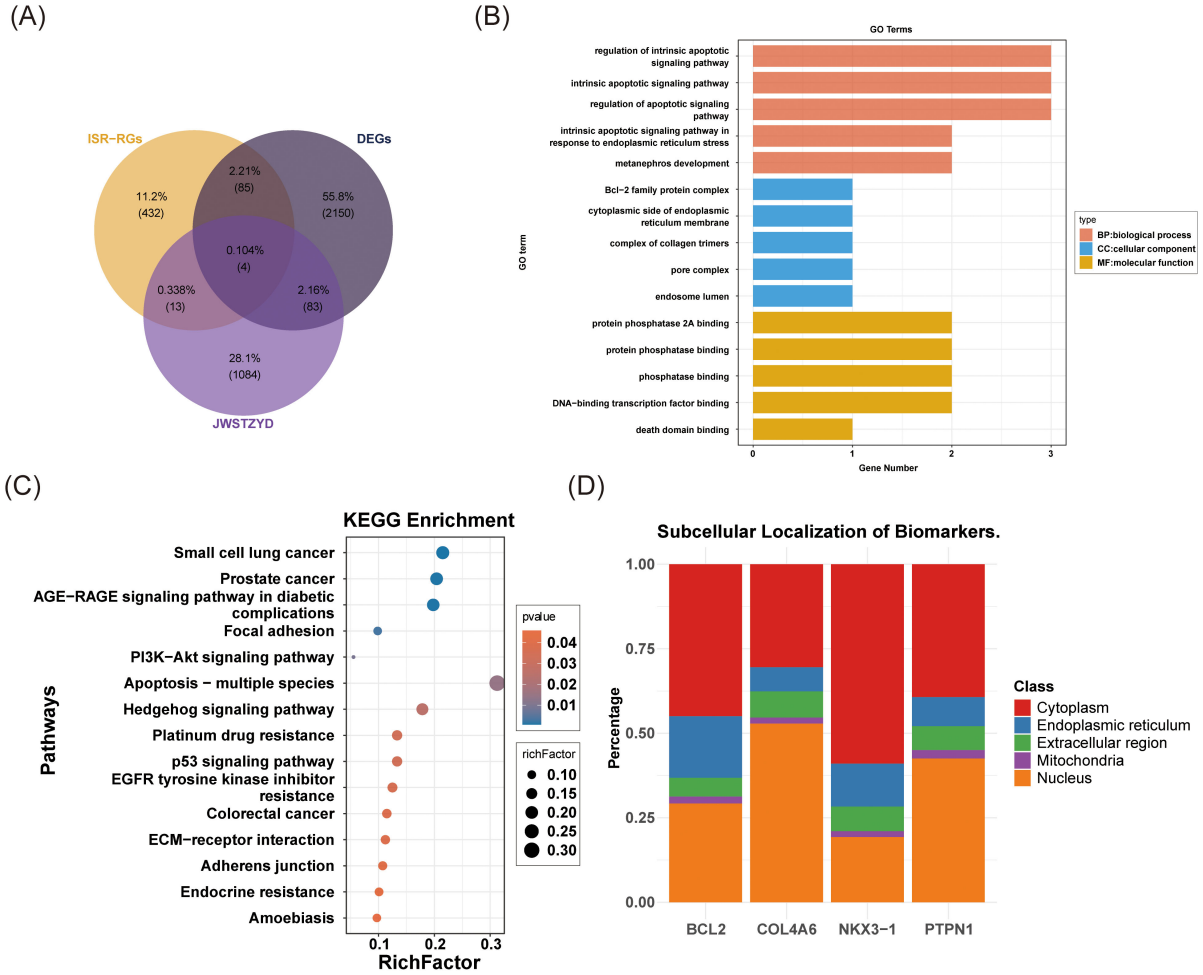
Screening, enrichment analysis, and cell localization analysis of OP candidate genes. **(A)** Venn diagram of the OP candidate genes. **(B)** GO enrichment analysis of OP candidate genes. **(C)** KEGG enrichment analysis of OP candidate genes. **(D)** Subcellular localization analysis of OP candidate genes.

Four candidate genes (*COL4A6*, *PTPN1*, *BCL2*, and *NKX3-1*) were selected based on the intersection of DEGs, ISR-RGs, and target genes of JWSTZYD (**Figure 3A**). The biological functions of these candidate genes were analyzed using GO, KEGG, and subcellular localization analyses. GO analysis identified 466 pathways, including 423 BPs, 13 CCs, and 30 MFs (P < 0.05) (**Supplementary Table 6**). The top 15 pathways, sorted by P-value, are displayed (**Figure 3B**). These included significant enrichment in 5 BPs (e.g., intrinsic apoptotic regulation, metanephros development), 5 CCs (e.g., collagen trimer complex, pore complex, endosome lumen), and 5 MFs (e.g., death domain binding, phosphatase binding). KEGG analysis revealed 15 significantly enriched pathways (P < 0.05) (**Supplementary Table 7**), including focal adhesion, hedgehog signaling, and p53 pathways (**Figure 3C**). Subcellular localization analysis showed that *COL4A6* and *PTPN1* were predominantly localized in the nucleus, while *BCL2* and *NKX3–1* were more concentrated in the cytoplasm (**Figure 3D**).

### 3.3 Identification of biomarkers

**Figure 4:**
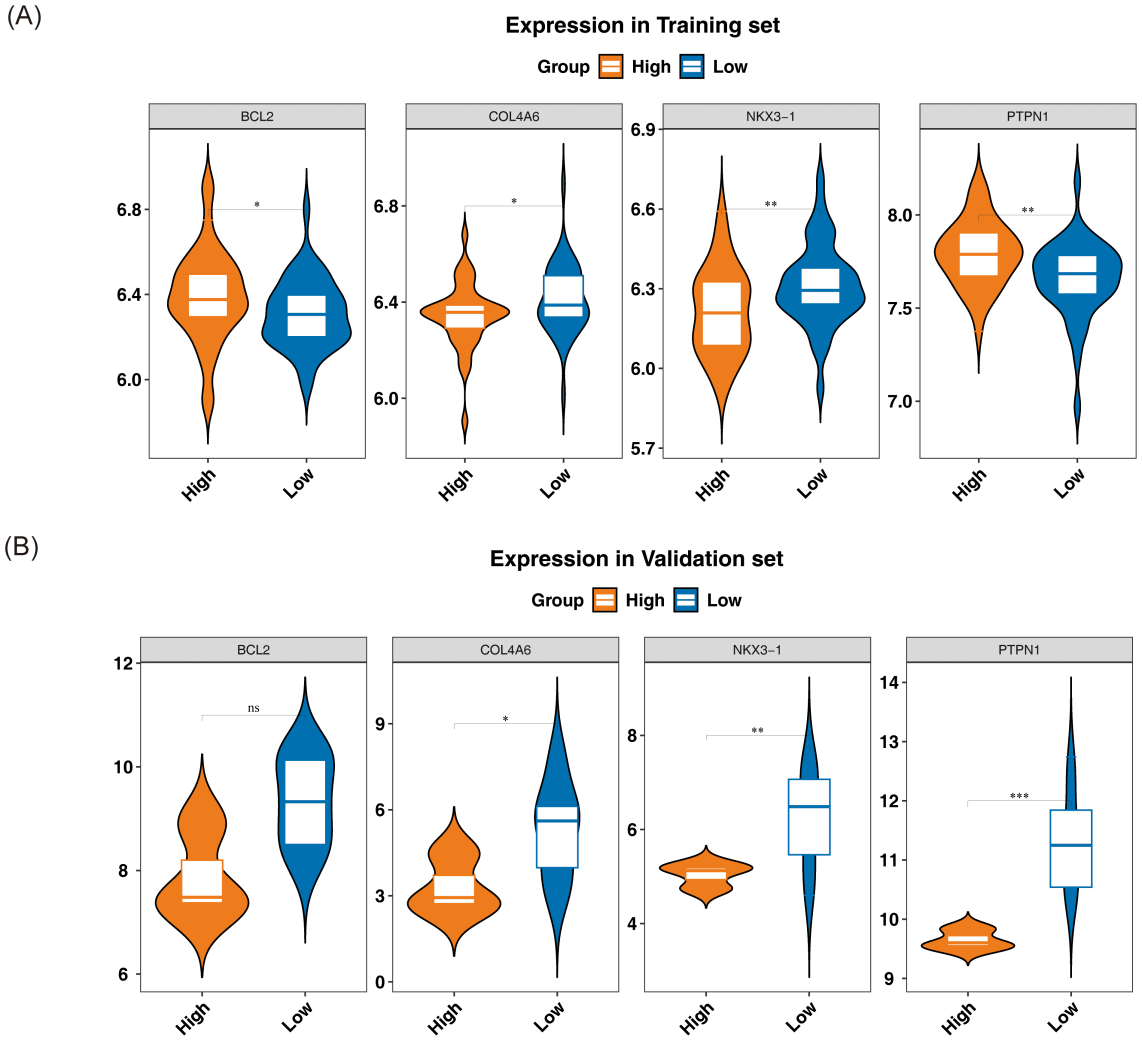
Relative expression levels of biomarkers. From left to right: *BCL2*, *COL4A6*, *NKX3-1*, and *PTPN1*. The horizontal axis represents different groups, while the vertical axis indicates expression levels. Yellow represents the normal bone mineral density group, and blue represents the low bone mineral density group. **(A)** Training set GSE56815. **(B)** Validation set GSE230665. In the figure, ns indicates not significant, and * indicates p < 0.05. ** represents p < 0.01; *** represents p < 0.001.

The expression levels of the candidate genes in GSE56815 and GSE230665 were analyzed to identify biomarkers. In GSE56815, the expression levels of *PTPN1* and *BCL2* were significantly lower in L-BMD samples compared to H-BMD, while *COL4A6* and *NKX3–1* were significantly higher in L-BMD than in H-BMD (P < 0.05) (**Figure 4A**). In GSE230665, *COL4A6*, *PTPN1*, and *NKX3–1* were significantly higher in L-BMD compared to H-BMD (P < 0.05) (**Figure 4B**). The expression trends of *COL4A6* and *NKX3–1* were consistent across both datasets, supporting their selection as biomarkers for further analysis.

### 3.4 Enrichment pathway analysis

**Figure 5:**
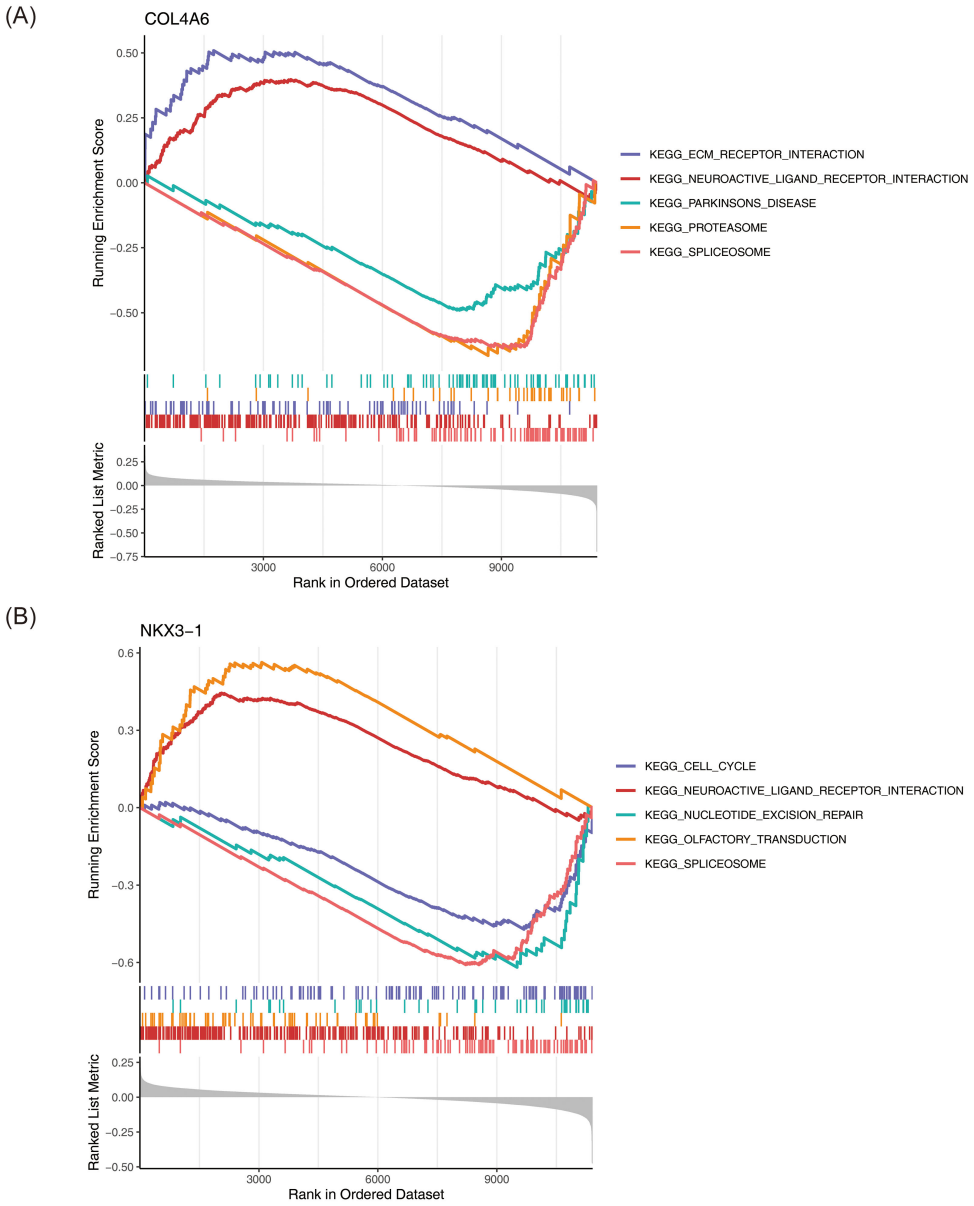
GSEA enrichment analysis of OP biomarkers. **(A)** *COL4A6*. **(B)** *NKX3-1*. This figure is divided into three parts: Part One: The top five line graphs are the line graphs of the Gene Enrichment Score. The vertical axis represents the corresponding Running ES. In the line graph, there is a peak, which is the Enrichment score of this gene set. The genes before the peak are the core genes under this gene set. The horizontal axis represents each gene under this gene set, corresponding to the vertical line similar to a barcode in the second part. Part Two: The section similar to barcodes is Hits, where each vertical line corresponds to a gene under the gene set. Part Three: The rank value distribution graph of all genes, with the vertical axis being the ranked list metric, that is, the value of the ranking quantity of this gene.

GSEA revealed that *COL4A6* was significantly enriched in 22 pathways, while *NKX3–1* was enriched in 23 pathways (P < 0.05) (**Supplementary Tables 8**, **9**). *COL4A6* was significantly associated with spliceosome, proteasome, and Parkinson’s disease pathways (**Figure 5A**), while *NKX3–1* was enriched in spliceosome, olfactory transduction, and cell cycle pathways (**Figure 5B**).

### 3.5 Immune cell analysis

**Figure 6:**
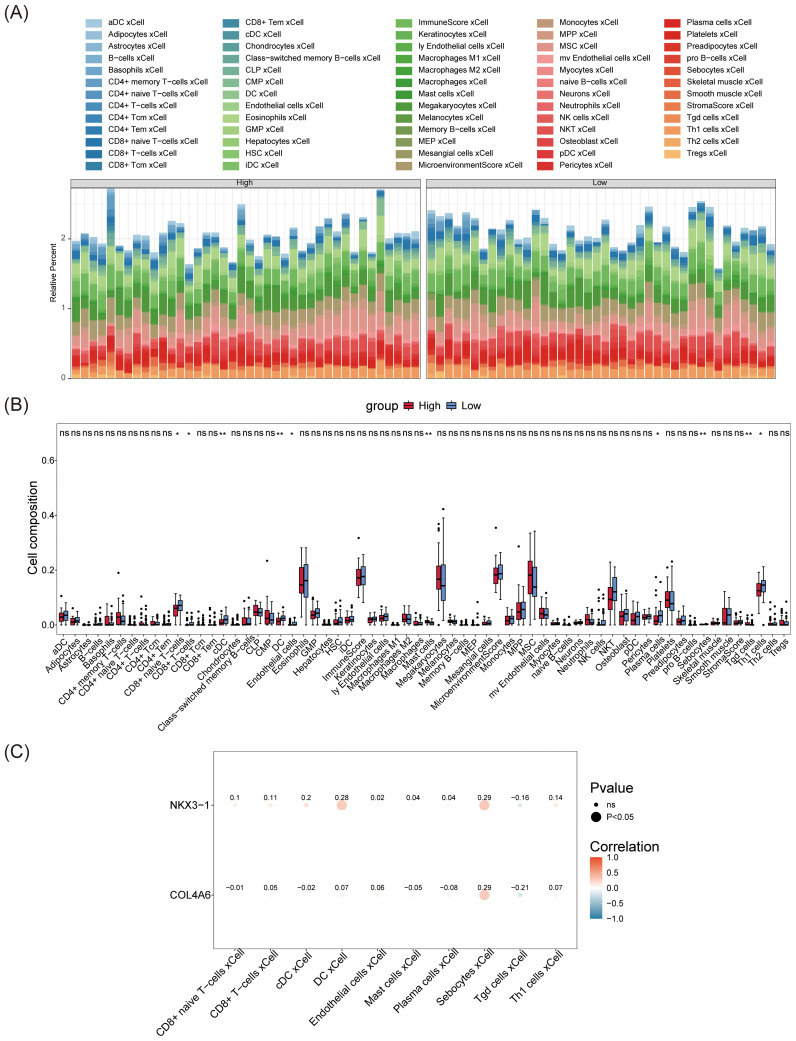
Immune infiltration analysis of OP biomarkers. **(A)** The immunoinfiltration abundance map of OP biomarkers. **(B)** Box plot of differences in immune cell abundance. The horizontal axis represents immune cells, and the vertical axis represents cell abundance. Red represents the control group, and blue represents the disease group. **(C)** Heatmap showing the correlation between OP biomarkers and differential immune cells. The horizontal axis represents differential immune cells, and the vertical axis represents biomarkers. The size of the dots reflects the significance of the response, and the color depth reflects the correlation.

The abundance of 64 immune cells between the L-BMD and H-BMD groups was analyzed using the Xcell algorithm (**Figure 6A**). Ten cell types showed significant differences between the two groups, including eight immune cells and two non-immune cells. The immune cells were CD8+ T cells, conventional dendritic cells (cDC), dendritic cells (DC), CD8+ naive T cells, mast cells (MCs), plasma cells (PCs), γδ T cells (Tgd), and CD4+ T cells (Th1), while the non-immune cells were endothelial cells (ECs) and sebocytes (**Figure 6B**). Correlation analysis between differential immune cells and biomarkers revealed that *NKX3–1* was weakly correlated with DC (cor = 0.28) and sebocytes (cor = 0.29), and *COL4A6* showed a weak correlation with sebocytes (cor = 0.29), while most other immune cells were not associated with the biomarkers (**Figure 6C**; **Supplementary Table 10**).

### 3.6 Construction of molecular regulatory network

**Figure 7:**
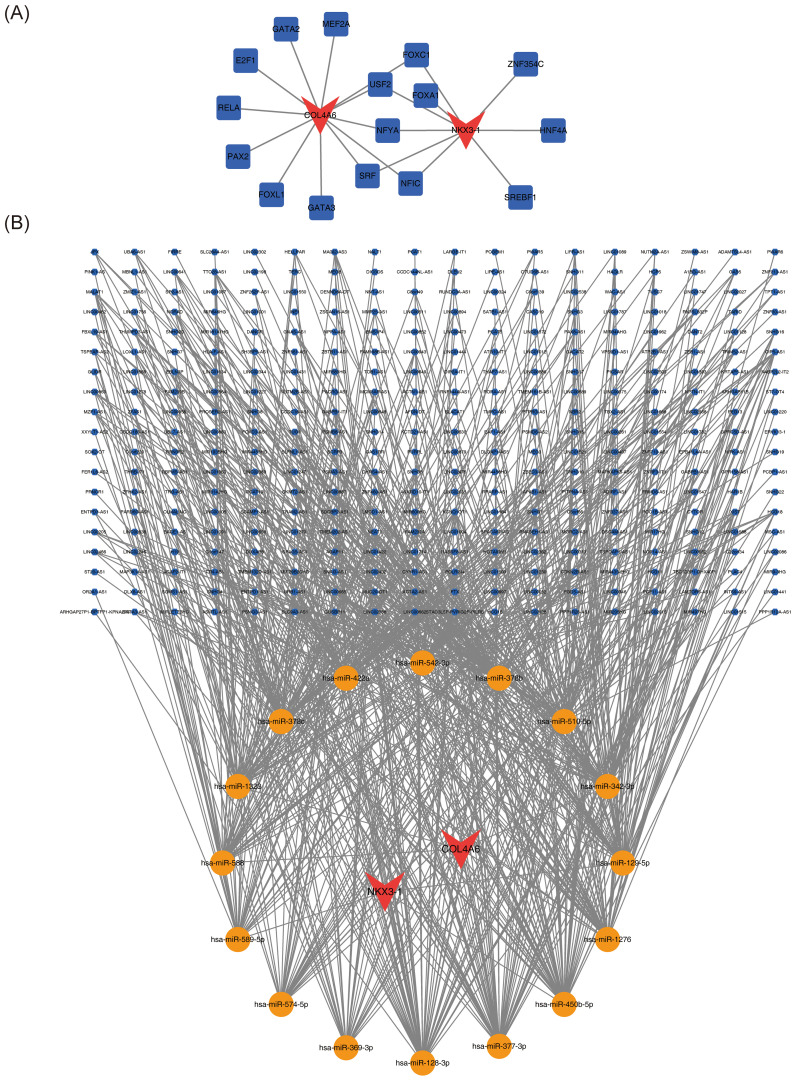
OP biomarker transcription factors and ceRNA regulatory network. **(A)** Regulatory network diagram of transcription factors and OP biomarkers. Red represents biomarkers, and blue represents transcription factors. **(B)** Molecular regulatory network diagram of OP biomarkers, with red representing biomarkers, yellow representing miRNAs, and blue representing lncRNAs.

Sixteen transcription factors (TFs), 26 miRNAs, and 342 lncRNAs were predicted through relevant databases. The TF-mRNA regulatory network comprised 12 interactions between the 16 TFs and the two biomarkers (**Figure 7A**). TFs such as FOXC1, USF2, NFYA, SRF, and NFIC were found to co-interact with *COL4A6* and *NKX3-1*. The lncRNA-miRNA-mRNA regulatory network revealed 748 relationships between the two biomarkers, 16 miRNAs, and 342 lncRNAs (**Figure 7B**).

### 3.7 Biomarker-drug docking

**Figure 8:**
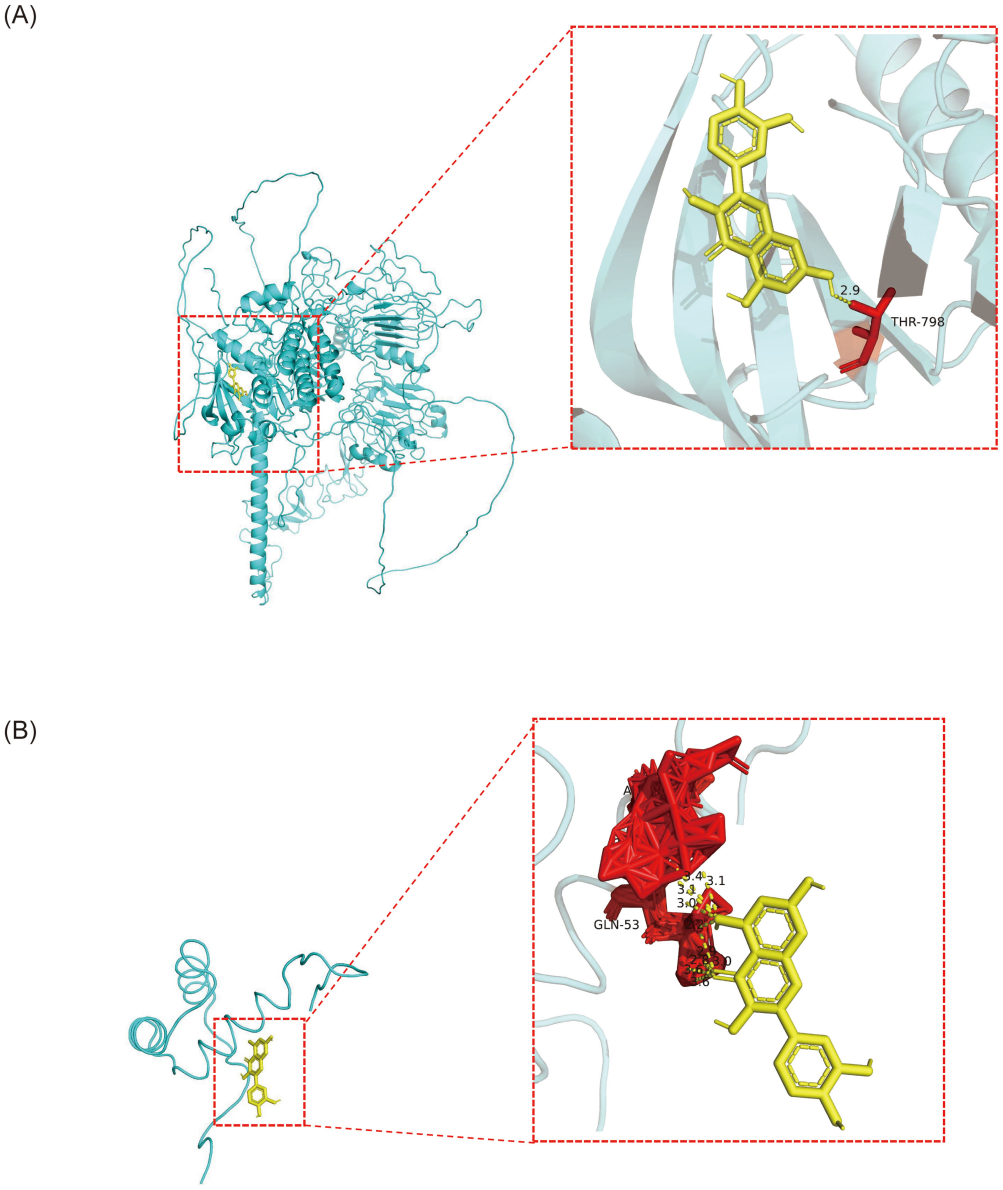
The molecular docking results of OP biomarkers *COL4A6* and *NKX3–1* with the drug small molecule quercetin. **(A)** Molecular docking result between the biomarker *COL4A6* and the small drug molecule quercetin. The light blue color represents the protein, yellow represents the small drug molecule, red represents amino acid residues that interact with them, and yellow dotted lines represent hydrogen bonds. **(B)** Molecular docking result between the biomarker *NKX3–1* and the drug small molecule quercetin. The light blue color represents the protein, yellow represents the small drug molecule, red represents amino acid residues that interact with them, and yellow dotted lines represent hydrogen bonds.

QUE, which interacted with both biomarker targets, was selected as a key drug for molecular docking with the biomarkers. The binding energies of QUE to *COL4A6* and *NKX3–1* were -6.3 kcal/mol and -15.7 kcal/mol, respectively, indicating strong binding affinity for both (**Figures 8A, B**; **Table 1**).

**Table 1:** Binding energy of biomarker protein docking with drug molecule.

Gene	Pro_ID	momle	Vina score
*COL4A6*	AF-Q14031-F1-v4	quercetin	-6.3
*NKX3-1*	2L9R	quercetin	-15.7

### 3.8 Validation of expression levels in clinical blood samples

**Figure 9:**
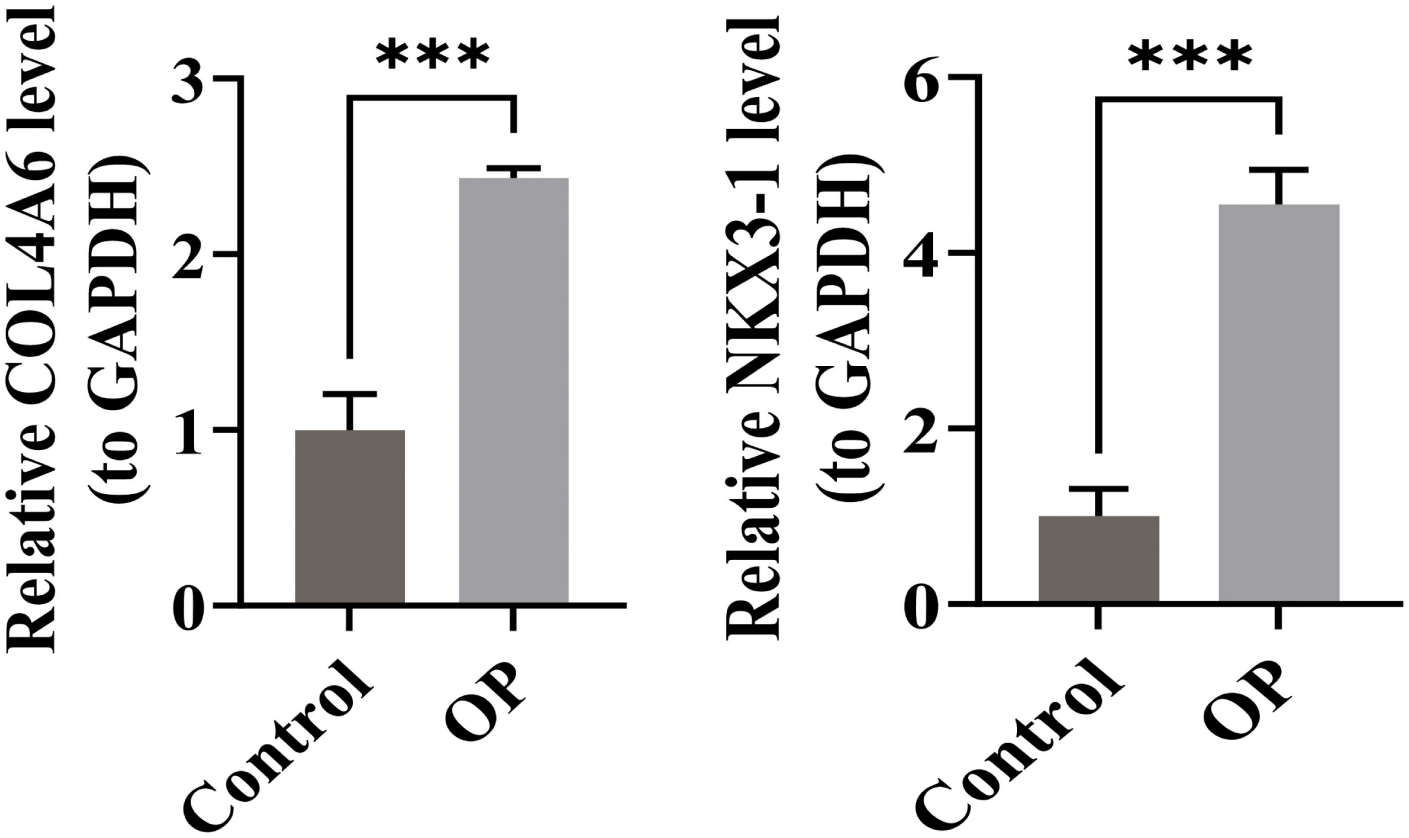
Comparison of RT-qPCR results for OP biomarkers COL4A6 and NKX3-1 in the control and OP groups.*** represents p < 0.001.

RT-qPCR was performed to validate the relative mRNA expression levels in OP and control groups. The results showed that the expressions of *COL4A6* and *NKX3–1* were significantly higher in the OP group compared to the control group (**Figure 9**). These RT-qPCR findings were consistent with the bioinformatics analysis results.

### 3.9 The mechanism framework of JWSTZYD in the treatment of osteoporosis

**Figure 10:**
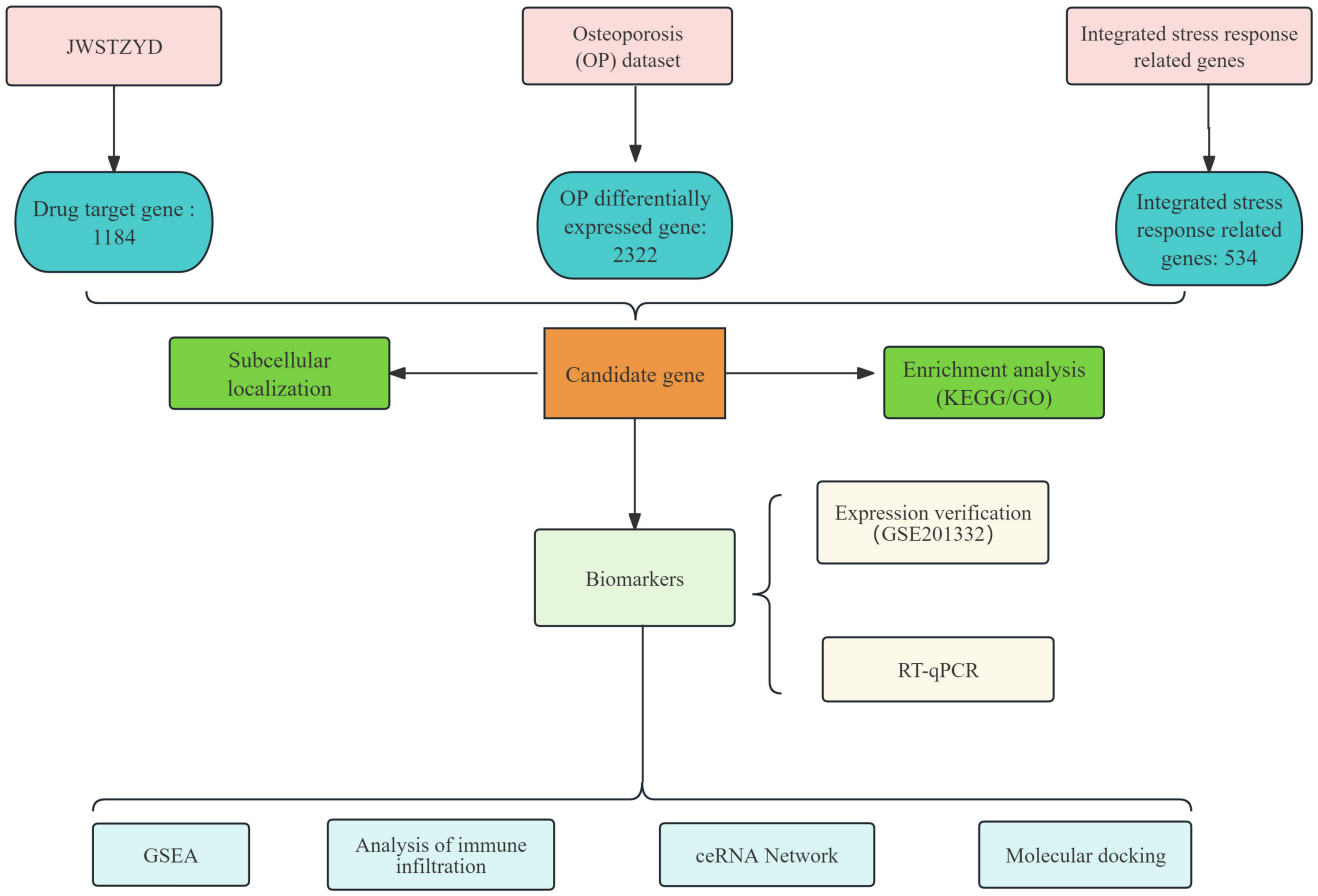
Schematic diagram illustrating the potential mechanism of JWSTZYD in treating osteoporosis via ISR-related biomarkers.

Combining the multi-faceted results of bioinformatics prediction and experimental verification, a mechanism model of the effect of JWSTZYD on OP was constructed (**Figure 10**). The model proposed that the therapeutic effects of JWSTZYD were primarily mediated through its key active ingredient, quercetin, which directly targeted the ISR-related core biomarkers *COL4A6* and *NKX3-1*. Modulation of these biomarkers orchestrated a broad downstream influence, encompassing the regulation of critical pathways such as the spliceosome and proteasome, coupled with a reconfiguration of the immune microenvironment evidenced by altered infiltration of dendritic cells, mast cells, and T lymphocytes. These concerted molecular and cellular events ultimately converged to ameliorate bone loss and restore osteogenic function, providing a novel theoretical foundation for JWSTZYD as a promising therapeutic strategy for osteoporosis.

## 4 Discussion

OP is a systemic skeletal disease characterized by bone mass loss and deterioration of bone microarchitecture (38). Previous studies have suggested that JWSTZYD may serve as an effective therapeutic agent in the treatment of OP. However, the specific targets and underlying mechanisms through which it exerts its effects are not fully understood and require further investigation.

This study screened 2,322 DEGs and 1,184 potential drug target genes for JWSTZYD. Four candidate genes were identified through the intersection of target genes, DEGs, and ISR-RGs, which were further filtered based on expression levels in OP and control samples. Two biomarkers, *COL4A6* and *NKX3-1*, were found to be associated with the ISR in JWSTZYD treatment for OP. Functional enrichment analysis revealed that JWSTZYD regulates multiple signaling pathways, including the spliceosome and neuroactive ligand-receptor interactions. Immune infiltration analysis also identified differential immune cells and two non-immune cells between OP and control groups, suggesting that the immune microenvironment may play a role in the pathogenesis of OP.

*COL4A6*, located on chromosome Xq22.3, encodes the collagen α6 chain and is associated with *COL4A5*. It is typically expressed in Bowman’s capsule, epidermis, and smooth muscle cells (39). Collagen, the most abundant protein in the extracellular matrix of most animals (40), plays a crucial structural role in tissue architecture, shape, and mechanical properties (41). A substantial body of research has confirmed the impact of collagen molecules on osteoblasts (42). In the mid to late stages of osteoblast differentiation, Col24a1 is activated through the binding of the CREB2-AP1 complex to upstream sequences (43). *COL4A6*, along with the proteins it codes (*COL4A4* and *COL4A5*), contributes to a structure involved in controlling vascularization during cartilage repair and maintaining cartilage homeostasis, with anti-angiogenic properties (44). Mengqi Guan et al. found that the aqueous extract of Eucommia leaves (an effective ingredient for treating OP) mildly increased *COL4A6* expression, promoting osteoblast proliferation, differentiation, mineralization, and bone formation and repair (45). Our study also confirmed the elevated expression of *COL4A6* mRNA in osteoporotic human bone. Therefore, *COL4A6* may serve as a critical marker for osteoblast differentiation.

*NKX3–1* is a TF that plays a pivotal role in the development and maintenance of various tissues (46, 47). It is widely recognized as a highly sensitive and specific marker for prostatic adenocarcinoma (48) and is recommended for evaluating cancers of unknown primary origin (49–51). Nimeka Ramanayake et al. suggested that NKX3.1 immunohistochemistry (IHC) might be sufficient for diagnosing mesenchymal chondrosarcoma (52). In mice, *NKX3–1* and *NKX3–2* play important roles in chondrogenesis (53). However, the correlation between *NKX3–1* and OP has not been explored, and further research is needed.

Regarding immune infiltration, significant differences were observed in the abundance of various immune and non-immune cells, including CD8+ T cells, CD8+ naive T cells, DCs, MCs, PCs, Tgd, Th1 cells, cDCs, ECs, and sebocytes (**Figure 5**). DCs are antigen-presenting cells (APCs) that play a pivotal role in activating the adaptive immune response. The impact of DCs on bone metabolism has been well-documented. Recent studies indicate that in an inflammatory environment, DCs can transdifferentiate into osteoclasts (OCs) (54), and in the presence of receptor activator of NF-kappaB ligand (RANKL), DCs contribute to bone resorption by differentiating into OCs (55). Moreover, DCs activate T cells, which secrete factors that drive bone remodeling (56). Activated DCs can develop into functional OCs upon interaction with T helper cells (57). Therefore, DCs may play a critical role in osteoclastogenesis. MCs, which are innate immune cells derived from bone marrow hematopoietic stem cells, have been found to induce osteoclastogenesis through the production of pro-inflammatory mediators (58, 59). In fracture healing studies, MCs regulate osteoclast activity (60). However, further research is needed to understand their role in bone metabolism and skeletal health. Inflammatory bone loss has also been linked to Th1 cells (61). The production of TNF-α by Th1 cells has been shown to promote osteoblast apoptosis and enhance osteoclastogenesis by increasing RANKL expression (62). CD8+ T cells regulate bone remodeling and inhibit osteoclastogenesis by secreting various soluble proteins (63). They form a negative feedback loop with osteoclasts, helping protect both the skeletal and immune systems during bone resorption (64). PCs, the final stage of differentiation of mature B lymphocytes (65), also regulate osteoclastogenesis *via* RANKL (66). Glycolysis in ECs triggers osteoblastic differentiation in bone mesenchymal stem cells *via* histone lactylation (67). In the same study, genetic experiments revealed that matrix metallopeptidase 9 provided by ECs is essential for cartilage resorption and directional bone growth (68). Additionally, further research is required to clarify the roles of γδ T cells and sebocytes in the context of OP.

The drug QUE was found to act on both biomarkers, *COL4A6* and *NKX3-1*, exhibiting stronger binding affinity in molecular docking with these biomarkers. QUE possesses antioxidant, anti-apoptotic, and anti-inflammatory properties, and has been reported to exert anti-OP effects (69). QUE promotes osteoblast-mediated bone matrix synthesis, enhancing the properties and stability of bone tissue (70). It also facilitates the proliferation and osteogenic differentiation of bone marrow-derived mesenchymal stem cells (BMSCs) by modulating the Wnt/β-catenin and BMP signaling pathways (71, 72). Additionally, QUE effectively inhibits osteoclastogenesis and activation through the modulation of the RANK/RANKL pathway, as well as the inhibition of the MAPK and NF-κB signaling cascades (73). By suppressing NF-κB pathway activation, QUE reduces oxidative stress-induced cell damage and bone loss (74, 75). It also activates the Nrf2 signaling pathway, mitigating the detrimental effects of oxidative stress on BMSCs and osteoblasts. In summary, QUE promotes bone formation, inhibits bone resorption, and provides anti-inflammatory and antioxidant effects by regulating key signaling pathways such as Wnt/β-catenin, BMP/TGF-β, RANK/RANKL, MAPK, NF-κB, and Nrf2. QUE is an important active ingredient in JWSTZYD that may exert therapeutic effects on OP through these mechanisms.

This study employed network pharmacology and bioinformatics analysis to identify two biomarkers (COL4A6 and NKX3-1) closely associated with the ISR pathway during JWSTZYD treatment for osteoporosis. Molecular docking validation confirmed their strong binding affinity with quercetin, a key active component of JWSTZYD, suggesting quercetin may serve as the fundamental substance through which JWSTZYD modulates the ISR pathway and improves bone metabolism. This discovery not only offers a novel perspective on elucidating the molecular mechanisms of OP but also provides potential targets and compositional rationale for the clinical application of JWSTZYD.

However, the study has some limitations. Firstly, the sample size in the GEO database dataset is relatively small. Secondly, while network pharmacology can predict the active ingredient-target associations of JWSTZYD, and molecular docking can assess the binding potential between components and targets, current research has yet to conduct actual experimental validation of the regulatory effects of active ingredients on pharmacodynamic-related genes. Finally, further experimental research is necessary to fully elucidate the role of these biomarkers and their underlying regulatory mechanisms in OP.

In order to further establish the functional role of biomarkers in ISR and bone metabolism regulation, as well as the therapeutic potential of quercetin, we plan to conduct the following studies in the future to address these shortcomings. Firstly, we will increase the sample size for OP and verify the differential expression of biomarkers using multicenter data to improve the reliability of the biomarkers. Secondly, we will use a combination of gene overexpression/silencing and surface plasmon resonance (SPR) and luciferase reporter gene experiments to verify quercetin’s binding activity and regulatory mechanism to the target. Thirdly, using the JWSTZYD gavage rat model, we will conduct multi-omics integration analysis on bone tissue to construct an ISR bone metabolism regulatory network. We will also establish a 3D osteoporosis organoid model to evaluate the therapeutic effect of the active ingredient combination. Additionally, the scope of active ingredient screening will be expanded and their interaction with other potential ingredients will be verified through *in vitro* and *in vivo* experiments. We will explore the regulatory mechanism of biomarkers in the ISR pathway in depth to systematically reveal the multi-target synergistic mechanism of JWSTZYD in the treatment of OP. This will provide a sufficient theoretical and experimental basis for its clinical translation.

In conclusion, this study identified *COL4A6* and *NKX3–1* as biomarkers linked to ISR in the treatment of OP with JWSTZYD, through NP and bioinformatics. Molecular docking results revealed QUE as a key active drug for these biomarkers. This finding not only highlights the important roles of *COL4A6* and *NKX3–1* in the mechanisms of OP and ISR, but also provides new drug targets and potential effective ingredients for treating OP with JWSTZYD, laying a solid foundation for further in-depth research and clinical application.

## Data Availability

The datasets presented in this study can be found in online repositories. The names of the repository/repositories and accession number(s) can be found in the article/**Supplementary Material**.
